# Post-contrast non-selective double inversion recovery imaging of the coronary arteries in patients with coronary allograft vasculopathy

**DOI:** 10.1186/1532-429X-13-S1-P241

**Published:** 2011-02-02

**Authors:** Sarah A Peel, Tarique Hussain, Michael Burch, Matthew Fenton, Andrew Taylor, Vivek Muthurangu, Gerald Greil, René M Botnar

**Affiliations:** 1 Division of Imaging Sciences, King's College London, London, UK; 2 Great Ormond Street Hospital for Children NHS Trust, London, UK

## Introduction

The uptake of gadolinium contrast agent in coronary walls may indicate metabolically-active atherosclerosis (Maintz et al, 2006 ) and therefore be useful in the setting of coronary allograft vasculopathy (CAV). The interpretation of inversion recovery (IR) images can be hampered by signal from tissues with longer T1 times (particularly the myocardium) as tissue suppression is T1 dependent and only optimal for one specific T1 species (e.g. blood). We sought to improve contrast-enhanced coronary vessel wall imaging using a novel non-selective double inversion recovery (NS-DIR) prepulse that provides signal suppression over a wide user-defined T_1_-range.

## Methods

The NS-DIR prepulse with two time delays, TI_1_ and TI_2,_ was implemented on a 1.5T MR scanner. TI_1_ and TI_2_ were optimized in MATLAB simulations by minimizing M_Z_^NS-DIR^ over a user-defined T_1_-range for a given heart rate.

A T_1_-phantom containing 11 T_1_-samples (T_1_-range=120ms-1730ms) was imaged with the IR and NS-DIR pre-pulses for simulated heart rates between 45 and 105bpm. For each prepulse, the signal-to-noise ratio (SNR) was calculated for each sample.

Nine patients who had undergone heart transplantation (ages=12-17y) were imaged ~20minutes after injection of 0.2ml/kg Gadobutrol using a 32-channel coil on a 1.5T MR Scanner. Firstly a coronary MRA was performed followed by a targeted, free-breathing, ECG-triggered, 3D-IR segmented gradient-echo (TFE) sequence along the right and left coronary arteries. Imaging parameters included spatial-resolution=1.25x1.25x3mm, TR/TE=3.5/1.4ms, FA=30° and the TI was chosen to null blood from a Look-Locker sequence. Subsequently, identical planes were repeated with the IR replaced by the NS-DIR pre-pulse with imaging parameters maintained. Inversion times TI_1_ and TI_2_ were set to suppress tissues with T1 values between 200-1400ms according to the patient’s heart rate. Imaging was performed every heartbeat at the mid-diastolic rest period.

## Results

**Figure 1:**
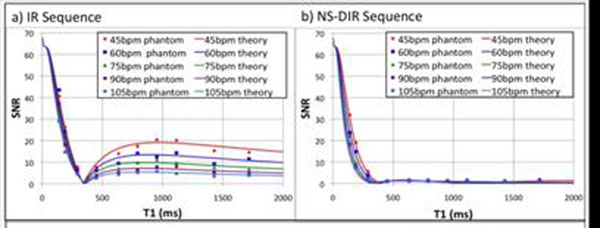
Simulated M_2_ values (solid lines) and phantom SNR values (data points) for a) the IR sequence (T1 set to null T1 species 340ms for different heart rates) and b) the NS-DIR sequence (TI_1_ and TI_2_ values optimized to minimize M_2_ for a range between 200 and 1400ms for different heart rates.) N.B. The theoretical Mz values have been scaled in order to display the data on the same graph.

Simulations and phantom studies show that the IR sequence (fig.1a) only nulls one T1 species whereas the NS-DIR sequence (fig.1b) achieves excellent signal suppression over the desired T_1_-range.

**Figure 2:**
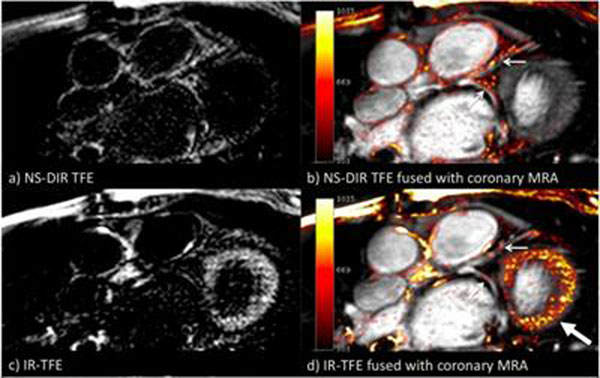
Post contrast imaging of the left coronary artery: a) NS-DIR TFE images, b) NS-DIR TFE images fused with coronary MRA, c) IR-TFE images and d) IR-TFE images fused with coronary MRA. Small arrows indicate enhancement of the coronary vessel walls and the large arrow indicates enhancement in the myocardium in the IR-TFE images.

Patient studies showed that the NS-DIR sequence (fig.2a) achieved simultaneous suppression of the blood and myocardium. Only the areas of contrast uptake are visible, which correspond to the path of the LCA (fig.2b). In contrast, interpretation of the IR images (fig.2c and fig.2d) was hampered by the bright signal in the myocardium.

## Conclusion

Simulations and phantom studies demonstrate that the NS-DIR sequence exhibits excellent tissue suppression over a wide T_1_-range. Preliminary patient data show improvement in contrast agent visualization in the coronary vessel walls in patients with CAV.

